# Association Between Daily Internet Use and Incidence of Chronic Diseases Among Older Adults: Prospective Cohort Study

**DOI:** 10.2196/46298

**Published:** 2023-07-17

**Authors:** Peiyi Li, Chenyang Zhang, Shuanliang Gao, Yanbo Zhang, Xiaolong Liang, Chengdi Wang, Tao Zhu, Weimin Li

**Affiliations:** 1 Department of Anesthesiology West China Hospital Sichuan University Chengdu China; 2 Laboratory of Anesthesia and Critical Care Medicine National-Local Joint Engineering Research Centre of Translational Medicine of Anesthesiology West China Hospital, Sichuan University Chengdu China; 3 The Research Units of West China (2018RU012)-Chinese Academy of Medical Sciences West China Hospital Sichuan University Chengdu China; 4 Institute of Hospital Management West China Hospital Sichuan University Chengdu China; 5 West China School of Medicine West China Hospital Sichuan University Chengdu China; 6 Chengdu University of Information Technology Chengdu China; 7 Department of Epidemiology and Population Health Albert Einstein College of Medicine Bronx, NY United States; 8 State Key Laboratory of Grassland Agro-Ecosystems College of Ecology Lanzhou University Lanzhou China; 9 Department of Respiratory and Critical Care Medicine West China Hospital Sichuan University Chengdu China; 10 Institute of Respiratory Health Frontiers Science Center for Disease-related Molecular Network West China Hospital, Sichuan University Chengdu China; 11 President's Office West China Hospital Sichuan University Chengdu China

**Keywords:** daily internet use, chronic disease, disease prevention, middle-aged and older adult, usage, internet use, technology use, chronic illness, association, incidence, middle age, older adult, gerontology, geriatric, aging, elder, national survey

## Abstract

**Background:**

Chronic disease incidence among the elderly is increasing, which is correlated with the acceleration of population aging. Evolving internet technologies may help prevent and provide interventions for chronic diseases in an accelerating aging process. However, the impact of daily internet use on the incidence of chronic diseases is not well understood.

**Objective:**

This study aims to investigate whether daily internet use by middle-aged and older adults may inhibit or promote the occurrence of chronic diseases.

**Methods:**

We included participants from the China Health and Retirement Longitudinal Study (CHARLS), a longitudinal survey of Chinese residents aged ≥45 years. We assessed 8-year data from wave 1 (June 2011-March 2012) to wave 4 (July-September 2018) in CHARLS. Data from wave 4 were used for a cross-sectional study, and data from all 4 waves were used for a longitudinal study. Self-reported data were used to track variables, including internet use, use frequency, and the incidence of different chronic diseases. Cox proportional hazards modeling was applied in the longitudinal study to examine the relationship between daily internet use and chronic diseases among middle-aged and older adults, while adjusting for sociodemographic characteristics and health behaviors. In addition, longitudinal data were used to analyze internet usage trends, and cross-sectional data were used to analyze the factors influencing internet use.

**Results:**

Among the 20,113 participants included in the longitudinal analyses, internet use increased significantly, from 2% to 12.3%, between 2011 and 2018. The adjusted model found statistically significant relationships between daily internet use and a lower incidence of the following chronic diseases: hypertension (hazard ratio [HR] 0.78, 95% CI 0.65-0.95, *P*=.01), chronic lung disease (HR 0.74, 95% CI 0.57-0.97, *P*=.03), stroke (HR 0.69, 95% CI 0.50-0.94, *P*=.02), digestive disease (HR 0.73, 95% CI 0.58-0.91, *P*=.005), memory-related disorders (HR 0.58, 95% CI 0.37-0.91, *P*=.02), arthritis or rheumatism (HR 0.60, 95% CI 0.48-0.76, *P*<.001), asthma (HR 0.52, 95% CI 0.33-0.84, *P*=.007), depression (HR 0.80, 95% CI 0.71-0.89, *P*<.001), and vision impairment (HR 0.83, 95% CI 0.74-0.93, *P*=.004). Moreover, our study also showed that with increasing frequency of internet use, the risk of some chronic diseases decreases.

**Conclusions:**

This study found that middle-aged and older adults who use the internet have a reduced risk of developing chronic diseases versus those who do not use the internet. The increasing prevalence of daily internet use among middle-aged and older adults may stimulate contemplation of the potential role of internet platforms in future research on chronic disease prevention.

## Introduction

The problem of an aging society has become a pervasive societal concern in both high- and low-income countries [1-3]. China, the second-largest economy in the world, is home to 1.4 billion people (18% of the worldwide population), whose population is rapidly aging [4,5]. The most important effect of this aging is the increase in patients with chronic diseases, because the worldwide pandemic of chronic diseases is significantly connected with population aging [6,7]. Chronic diseases, which include hypertension, diabetes, depression, and other illnesses [8], are defined by the Centers for Disease Control and Prevention as ailments that persist for a year or more and need continuous medical care, restrict everyday activities, or both [9]. Worldwide, chronic diseases are becoming the leading contributor to the disease burden among older people [10]. Therefore, effective prevention of and interventions against chronic diseases in the elderly population have been advocated and studied to address this burden, not only in terms of health care, but also in terms of lifestyle [11-13].

Internet technology has shown promise for the provision of appropriate and prompt health care support for people with chronic conditions [14-16], including data collection, doctor-patient contact, patient monitoring, and health education [17,18]. For instance, internet-based telemedicine technology may be used for monitoring and disease intervention outcomes in patients with heart failure and diabetes, therefore lowering mortality and hospital admissions [19]. However, some studies have shown that internet use by older people, particularly excessive or addictive use, may be hazardous to their health [20,21]. A person in poor health, especially one who is ill, may not benefit from using the internet, because acquiring medicines or advice without actively accessing health care would not help their condition [22]. Moreover, unrestrained and excessive internet use has been linked to poor eyesight, sleep loss, emotional discomfort, and memory problems, as well as decline in social involvement, in older people, all of which have an effect on their physical and mental health [20,23-27].

Even though the aforementioned research has not yet generated a unified conclusion, older populations with chronic diseases and impairments may be able to access information about chronic disease management that will assist them in deciding whether and how to use the internet in their daily lives. In contrast, research on elderly people without chronic diseases is limited. The incidence of chronic diseases increases with age [28], reaching 62.3% among people older than 65 years [29]. Therefore, the early prevention of chronic diseases in older people is increasingly important, and the evolving internet technology may provide a solution to this issue [30]. The use of eHealth [31], mobile health (mHealth) [32], and telehealth [33] offers an opportunity to assist elderly people in improving their overall health and may help prevent chronic diseases [34-36]. Specifically, for some high-risk groups, for example, those with impaired glucose tolerance, overweight or obesity, dyslipidemia, or uncontrolled blood pressure, using telehealth may alert them to monitor their health status data [36]. eHealth interventions have also been shown to have a positive effect on improving timely access to medical services and increasing patient satisfaction [37]. Notably, elderly individuals are more likely to use the internet for routine daily activities, such as watching videos and staying informed about current events, rather than for internet-based health care delivery, which often requires higher levels of digital health literacy [5,34,38]. Although daily internet use among healthy older adults is reflective of real-world scenarios, there is a paucity of research examining the effects of such daily use on this population’s health. There is evidence that using the internet may be effective in increasing physical activity, which may help prevent obesity, heart disease, hypertension, diabetes, and even premature death in elderly individuals [35,39]. Internet use may also provide elderly people with convenience, leisure, information, and social interaction, which helps them promote social connectedness, reduce social isolation, obtain social support, accumulate social capital, and, thus, reduce the occurrence of depression [40,41]. Frequent internet use also has the potential to mitigate the detrimental consequences of social isolation on mental health in middle-aged and older adults, with lower feelings of loneliness and higher life satisfaction [42,43]. Although previous studies have demonstrated that internet use can have substantial effects on health outcomes of elderly individuals, the majority of these investigations focus on health-related internet use rather than daily internet use specifically. Therefore, further research is needed to determine whether and to what extent daily internet use, as well as the frequency of use, can confer similar health benefits for healthy older individuals.

The number of Chinese netizens, who are defined as Chinese citizens who use the internet for at least 1 hour per week, reached 1.067 billion as of December 2022, ranking first in the world; 30.8% of them are more than 50 years old [44]. Daily online activities of senior Chinese people include entertainment as well as social connection and communication, such as instant chatting, network news, and video viewing [45,46]. With the rapid increases in both population aging and internet use among older people in China, these internet behaviors are increasingly becoming everyday activities among middle-aged and older adults. It would be of great practical value to investigate whether daily internet use may inhibit or promote the occurrence of chronic diseases in older people in order to improve the health of the entire population.

Our research includes a survey of individuals aged ≥45 years. This study used data from the 4 waves of the China Health and Retirement Longitudinal Study (CHARLS) survey [47] to address 3 knowledge gaps: (1) the sociodemographic characteristics of daily internet use among middle-aged and older adults in China, (2) the changing trends in internet use over the past decade, and (3) the hazard ratios (HRs) of the occurrence of chronic diseases associated with internet use and high-frequency internet use compared with nonuse among healthy middle-aged and older adults.

## Methods

### Data and Sampling

Data from the 4 waves of the CHARLS survey, which started in 2011 (wave 1) and was repeated in 2013, 2015, and 2018 (waves 2, 3, and 4), were used in this study. CHARLS, a longitudinal survey of Chinese residents aged ≥45 years, used multistage sampling to sample community inhabitants’ health, social, and economic conditions. All data were collected by the CHARLS interviewers through face-to-face computer-assisted personal interviews (CAPIs). The interviewers were trained at Peking University by CHARLS staff members, and the interviews took place in respondents’ homes using CAPI technology [47]. After follow-up, 25,589 adults constituted the longitudinal cohort. For each step of this investigation, participants were interviewed individually. According to the World Health Organization, older adults are defined as those over 60 years old, while those aged 45-59 years are middle-aged adults [48]. By 2021, the population aged 45-59 years accounted for 24.5% of the total population in China, and the population over 65 years old accounted for 18.9% [4]. Among these groups, the prevalence of chronic diseases was 62.3% in people over 65 years old and was also high in people aged 45-54 years and 55-64 years, at 31.3% and 48.4%, respectively [29]. Therefore, considering that the middle-aged population is in a transitional stage of aging and also has a high incidence of chronic diseases [49], we also included people aged 45-59 years, in addition to older adults aged ≥60 years, for a more comprehensive data analysis.

**Figure 1:**
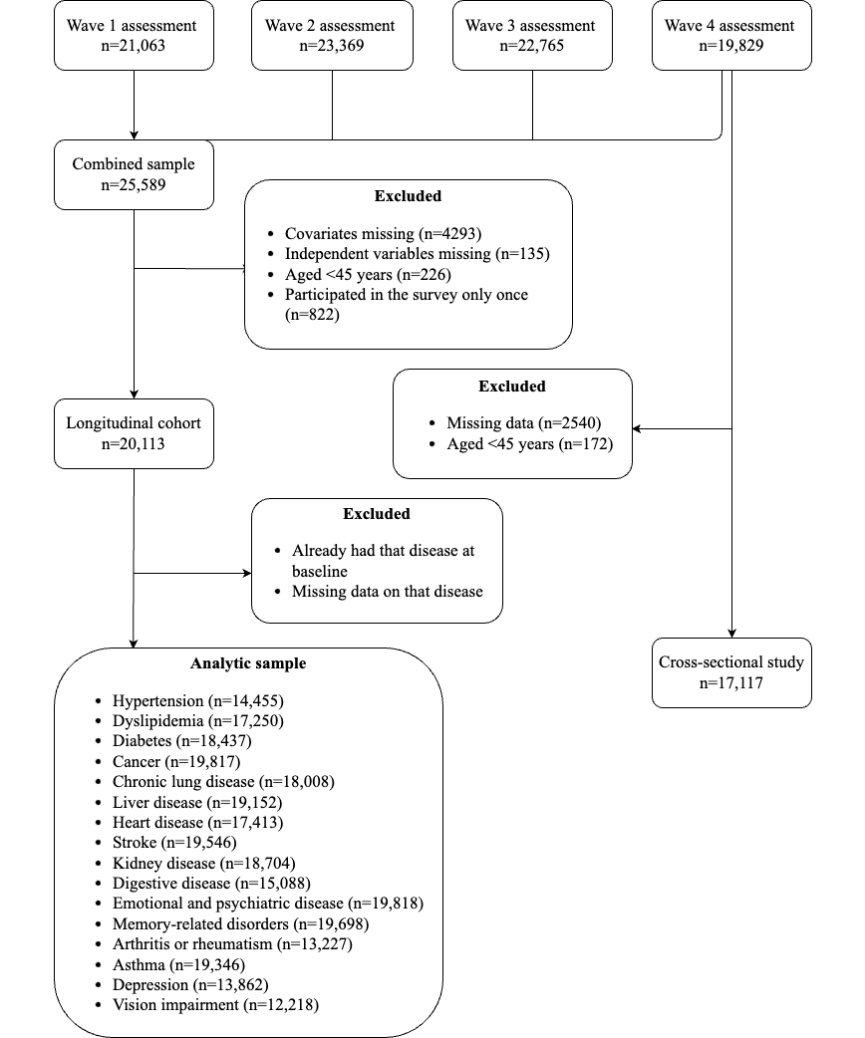
The study flow.

To address this first knowledge gap, we included wave 4 data of the CHARLS survey, which ended in 2018, for a cross-sectional study. After eliminating missing data (n=2540, 12.8%) and participants who were 44 years or younger (n=172, 0.9%), 17,117 individuals were included to determine the sociodemographic characteristics of middle-aged and older internet users. Longitudinal data, including 4 waves, were used to address the second and third knowledge gaps. First, after removing the data that did not meet the inclusion requirements (Figure 1), there were 20,113 participants remaining in the survey whose usage behavior was used to determine the trends in internet use over the past decade. For the third study point, we performed separate risk assessments for each chronic disease to determine the association between daily internet use and chronic diseases. Consequently, on the basis of these 20,113 individuals, we analyzed the longitudinal data of each disease and excluded baseline disease carriers. For example, in the case of hypertension, baseline hypertension (n=5632, 28%) and missing data (n=26, 0.1%) were removed before analysis, leaving 14,455 people for the next analysis. Figure 1 depicts the research process and chronic diseases covered.

### Measures

#### Sociodemographic Factors

Sociodemographic factors, including gender, education, nationality, work status, recruitment age (in 10-year increments), resident area, marital status, and health insurance, were extracted from each wave of follow-up surveys.

#### Health Behaviors

Information about health behaviors included (1) smoking status (yes and still smoked=1, never smoked/quit=0), (2) drinking status (more than once a month or less than once a month=1, none=0), (3) restless sleep (rare=1, some or a little=2, occasionally=3, mostly or all the time=4), and (4) activities of daily living (ADLs), which were assessed using the Activity of Daily Living Scale developed by Lawton and Brody [50], yielding a total score of 6-24, and divided into 5 categories (see Table S1 in Multimedia Appendix 1) [51,52]. The reliability coefficient of these items in terms of Cronbach α is .89 [53]. In the statistical analysis, the ADL score was classified as either “impaired” or “unimpaired” due to the small number of people with severe disabilities.

#### Daily Internet Use and Frequency

Participants were asked whether they had used the internet in the preceding month (yes=1, no=0). If a participant’s answer was yes, they were then questioned about how often they had used the internet in the previous month (almost daily=3, almost every week=2, not frequently=1). In this study, “internet use” refers to daily internet use, including but not limited to chatting, viewing news, watching movies, playing games, managing finances, and other things. To some extent, this is in line with the usage habits of middle-aged and older adults [38,44].

#### Chronic Disease Status

We collected interview data on chronic disease status using the self-reported question “Have you been diagnosed with [chronic condition] by a doctor?” Each participant’s disease status included a total of 14 noncommunicable chronic diseases: hypertension, dyslipidemia, diabetes, cancer (excluding minor skin cancers), chronic lung disease, liver disease (except fatty liver, tumors, and cancer), heart disease (including heart attack, coronary heart disease, angina, congestive heart failure, or other heart problems), stroke, kidney disease (except tumors or cancer), digestive disease (except tumors or cancer), emotional and psychiatric disease, memory-related disorders, arthritis or rheumatism, and asthma.

Depression was measured with 10 questions from the Center for Epidemiologic Studies Depression Scale (CES-D10) [54], a valid, reliable, and useful mental health measure (Cronbach α=.78-.79) for the middle-aged and older population in China [55-57]. The participants were asked the 10 questions regarding their feelings and behaviors over the previous week. Each response ranged from 0 to 3, providing a total score ranging from 0 to 30. Based on prior research, a cut-off score of 10 was generally selected, resulting in high specificity in older samples. Thus, we used a cut-off score of 10 to separate people with depressive symptoms from those without (Table S1 in Multimedia Appendix 1) [55,58,59].

Additionally, vision impairment (VI) was measured with 6 questions about eyesight. In this study, middle-aged and older adults were classified as having VI if they reported any vision problems (Table S1 in Multimedia Appendix 1) [60-62].

### Statistical Analysis

R version 4.1.2 (R Foundation for Statistical Computing) was used for all statistical calculations, and 2-tailed *P*<.05 was considered statistically significant. We substituted surrounding follow-up data for each participant in place of the missing data to ensure that the fraction of missing variables was less than 5% [63]. Case-weighted percentages were generated to describe sociodemographic factors and level distributions among participants.

In the cross-sectional study, categorical variables were presented as numbers and percentages and compared using chi-square tests and the Fisher exact test. For ordinal categorical variables, the Mann–Whitney *U* test and Kruskal–Wallis tests were used to compare sociodemographic characteristics between groups. Binary logistic regression and ordinal logistic regression were used for multivariate analysis of internet use and internet use frequency, respectively. In logistic regression analysis, first, the variables having statistical significance in the univariate analysis were included, then stepwise regression was used to find and eliminate multicollinearity, and finally the ideal model was produced. Ordinal logistic regression passed the test of parallel lines. Odds ratios (ORs) with 95% CIs were used to analyze the relationship between internet use and individual characteristics.

In the trend analysis using longitudinal data, chi-square tests and the Fisher exact test were used to compare the prevalence and frequency of internet use by gender.

In the longitudinal population, daily internet use and chronic disease incidence were examined using a Cox proportional hazards model, with HRs and 95% CIs. Three models were created based on the degree of adjustment: model 1 was a univariate model of internet use; in model 2, sociodemographic factors, such as gender, education, nationality, work status, recruitment age, resident area, marital status, and health insurance, were considered; and model 3 was then further modified to account for health behaviors, including smoking status, drinking status, restless sleep times, and ADLs. Follow-up time (in years) from baseline was used as the timeline, and the endpoint for each participant was either the date of the last follow-up or the date of disease manifestation, whichever came first.

### Ethical Considerations

CHARLS was a survey approved by the Ethical Review Committee of Peking University (approval number IRB00001052–11015), and the study data were anonymous. Each participant provided signed informed consent at the time of participation. There was no requirement for additional ethics approval for approved data users.

## Results

### Participant Details

Among wave 4 participants in our study, 14,805/17,117 (86.5%) did not use the internet, while 2312/17,117 (13.5%) did. Details of the characteristics of the study population are presented in Table 1. The binary logistic regression model showed that women, those with higher education, those not working, the relatively young, urban residents, nonsmokers, alcohol drinkers, those rarely experiencing restless sleep, and those with unimpaired ADLs were more likely to use the internet in daily life (Table 2). On this basis, we further conducted univariate analysis and ordinal logistic regression analysis according to the frequency of internet use (Table S2 in Multimedia Appendix 1). Ordinal logistic regression analysis showed that middle-aged and older adults were more likely to use the internet more regularly if they were women, well-educated, relatively young, living in an urban area, not smoking, drinking, and rarely experiencing restless sleep (Table 2).

**Figure 2:**
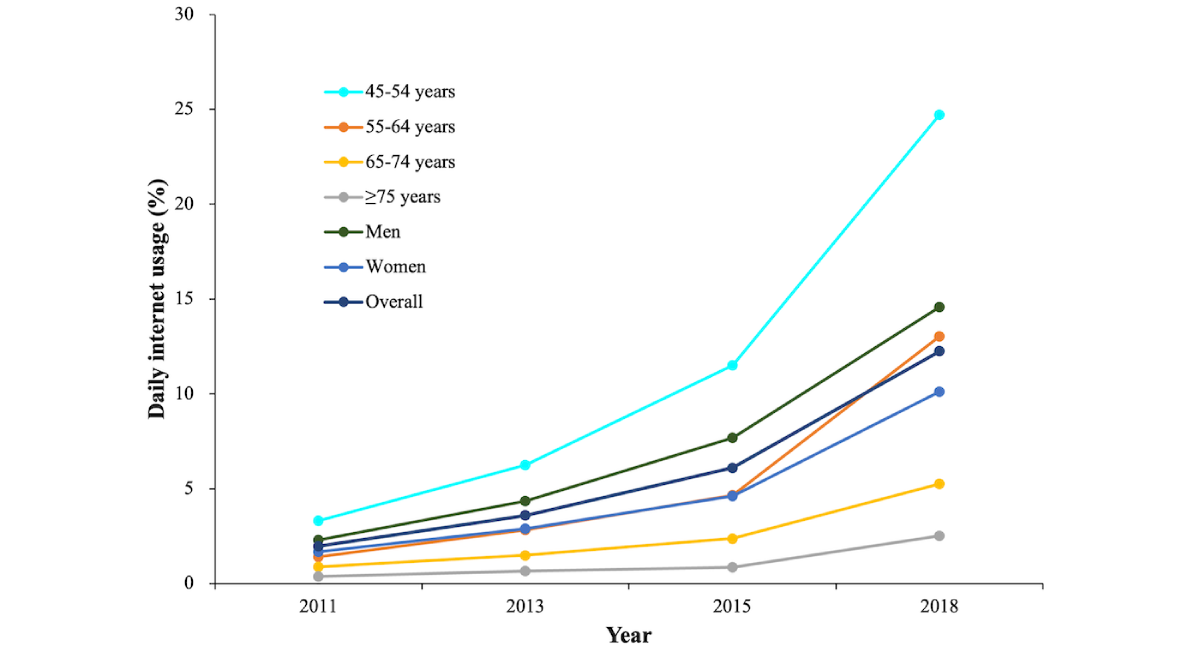
Daily internet usage among middle-aged and older adults: CHARLS 2011-2018. The y axis is the percentage of people who used the internet in their daily lives at each follow-up visit. CHARLS: China Health and Retirement Longitudinal Study.

**Figure 3:**
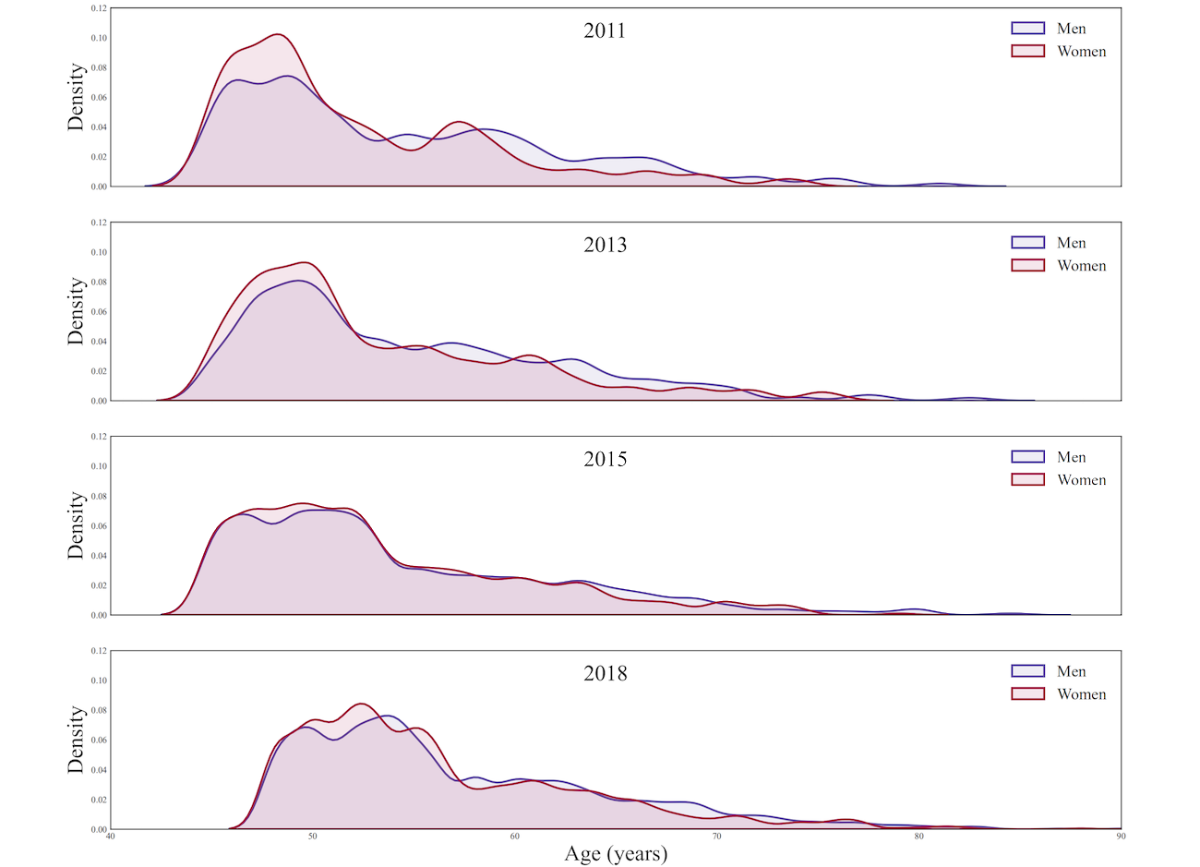
Density plot for age distribution of daily internet use among middle-aged and older adults.

**Figure 4:**
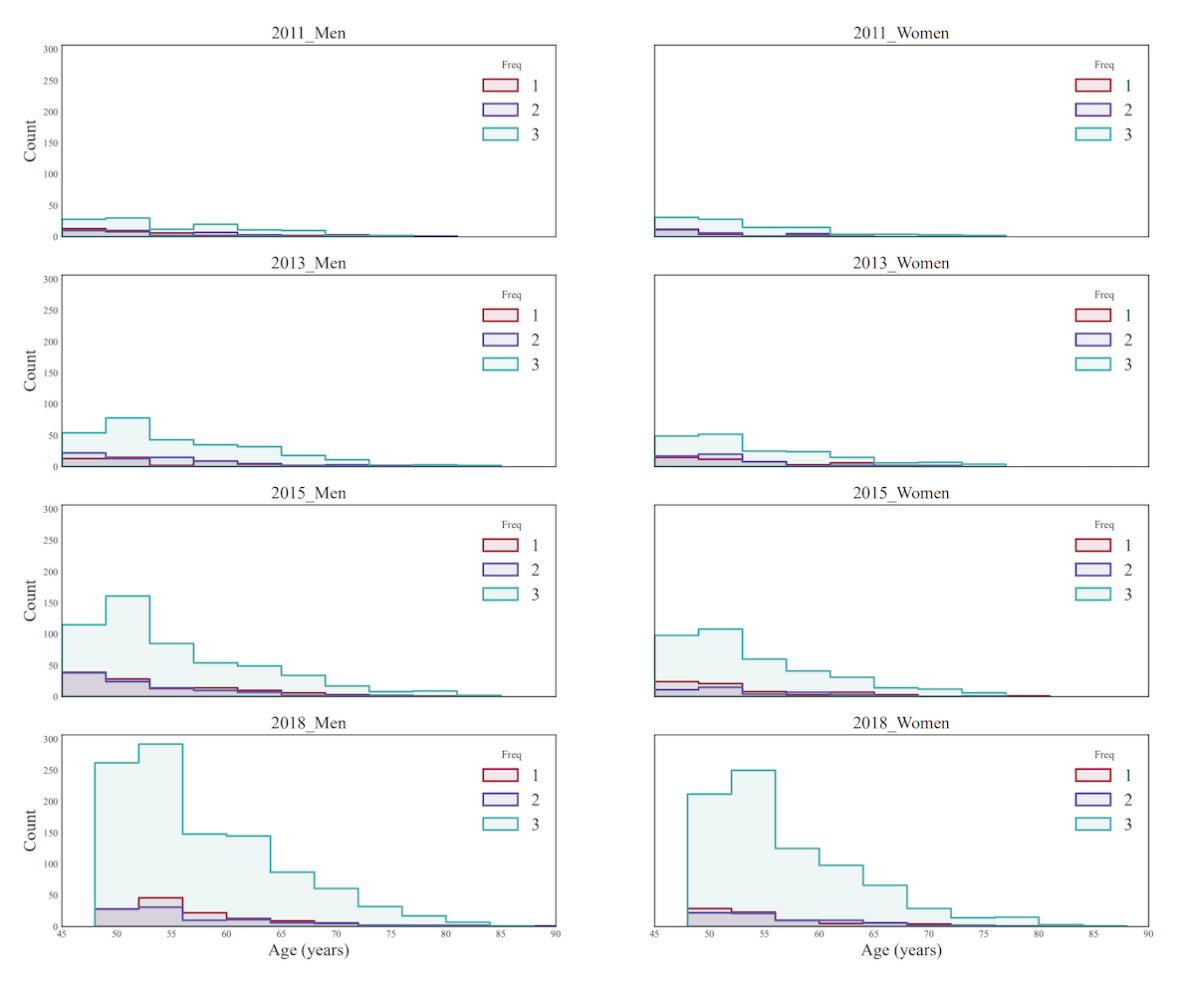
Histogram for frequency of daily internet use among middle-aged and older adults. Freq: frequency.

Overall, internet use has grown rapidly in recent years, from 2% in 2011 to 12.3% in 2018. In this study, the increase was larger for men (12.3%) than for women (8.4%). Across all age categories, there was significant growth in internet use. When compared to other age groups, individuals aged 45-54 years had the biggest rise in internet use, up by 21.4%, followed by those aged 55-64 years, while those aged over 75 years had the smallest growth (Figure 2). Figure 3 is a density plot showing the distribution of internet use in each age group for men and women over the past decade. For both men and women, the 45-54-year age group was the largest group of middle-aged and older adults using the internet, and with ascending age, the internet usage rate steadily fell. Figure 4 is a histogram showing the distribution of the internet use frequency in the past decade for each age group of men and women. As can be seen from the change in the frequency ratio in the past 10 years, the frequency of internet use among middle-aged and older adults steadily rose and was sustained at a high frequency. This rising tendency was more pronounced in men. Even though there were relatively fewer older adults using the internet—especially those aged over 75 years—this group had the largest proportion of internet users who used the internet almost daily, which means they had a higher frequency of internet use than the relatively younger middle-aged adults in recent years. Figure S1 in Multimedia Appendix 1 shows that over time, an increasing number of age groups exhibited gender differences in internet use, with men having slightly higher rates of internet use than women. In 2011, there was a gender difference only among participants aged 55-64 years, but by 2015 and 2018, there were gender differences in all 4 age groups. Similarly, the change in use frequency also shows that the gender difference in internet use has become increasingly obvious over time, with men using the internet slightly more frequently than women (Figure S2 in Multimedia Appendix 1). Especially in the age group of 45-54 years, there were gender differences in the 3 frequency groups in 2015 and 2018.

**Figure 5:**
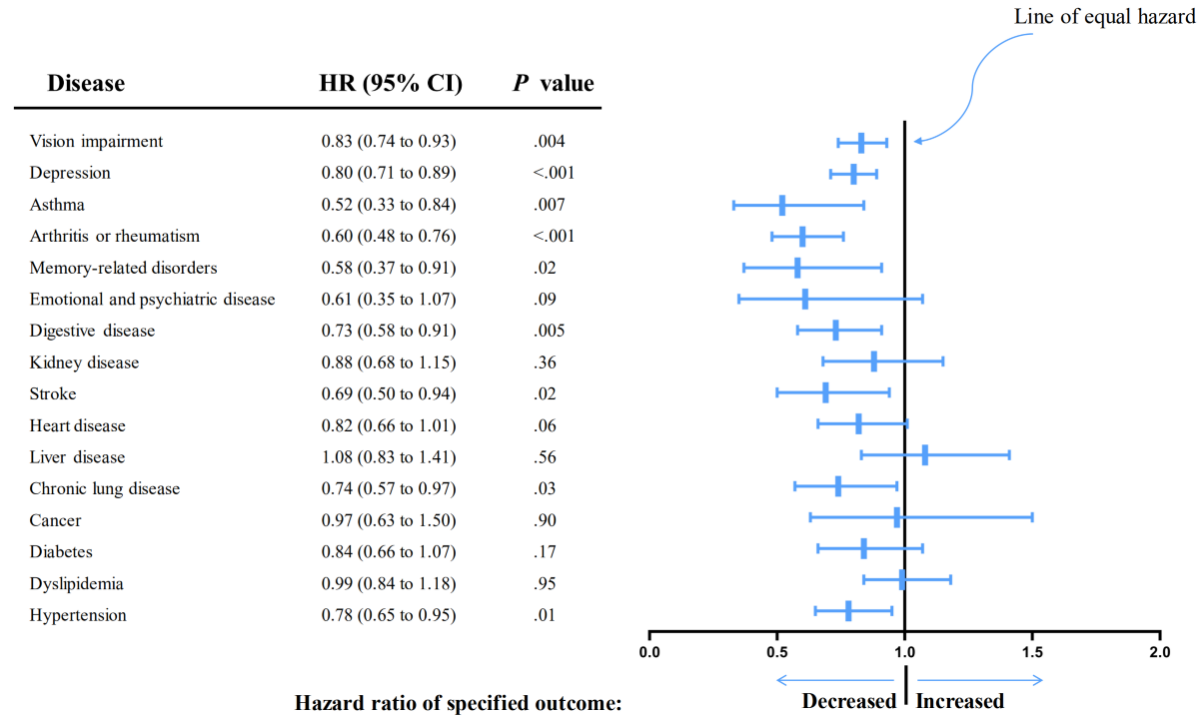
Forest plot for association between chronic diseases and daily internet use. HR: hazard ratio.

Interestingly, Cox proportional hazards modeling revealed that using the internet was associated with lower rates of most chronic diseases. Figure 5 shows that in the adjusted model 3, daily internet use was associated with a reduced risk of hypertension (HR 0.78, 95% CI 0.65-0.95), chronic lung disease (HR 0.74, 95% CI 0.57-0.97), stroke (HR 0.69, 95% CI 0.50-0.94), digestive disease (HR 0.73, 95% CI 0.58-0.91), memory-related disorders (HR 0.58, 95% CI 0.37-0.91), arthritis or rheumatism (HR 0.60, 95% CI 0.48-0.76), asthma (HR 0.52, 95% CI 0.33-0.84), depression (HR 0.80, 95% CI 0.71-0.89), and VI (HR 0.83, 95% CI 0.74-0.93) compared to internet nonuse. Model 2 showed that in addition to these chronic diseases, internet use also was associated with decreased incidences of heart disease as well as emotional and psychiatric disease (Table S3 in Multimedia Appendix 1).

**Figure 6:**
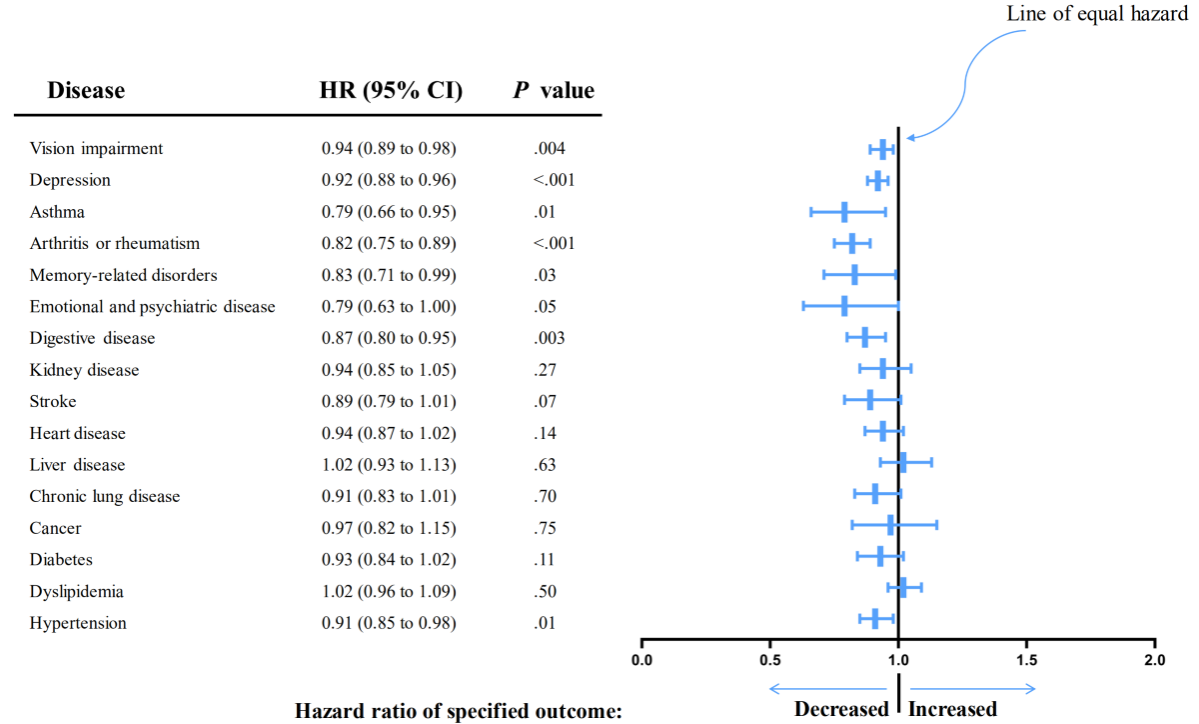
Forest plot for association between chronic diseases and daily internet use frequency. HR: hazard ratio.

The risk of chronic diseases, such as hypertension (HR 0.91, 95% CI 0.85-0.98), digestive disorders (HR 0.87, 95% CI 0.80-0.95), memory-related disorders (HR 0.83, 95% CI 0.71-0.99), arthritis or rheumatism (HR 0.82, 95% CI 0.75-0.89), asthma (HR 0.79, 95% CI 0.66-0.95), depression (HR 0.92, 95% CI 0.88-0.96), and VI (HR 0.94, 95% CI 0.89-0.98), also decreased as internet use frequency increased in the adjusted model 3 (Figure 6). Model 2 added stroke, chronic lung disease, and emotional and psychiatric diseases (Table S4 in Multimedia Appendix 1).

**Table 1:** Characteristics of participants (N=17,117) by internet use.

Characteristics and variables			Internet use (n=2312), n (%)		Internet nonuse (n=14,805), n (%)
**Gender; *P*<.001**					
	Men	1287 (55.7)		6902 (46.6)	
	Women	1025 (44.3)		7903 (53.4)	
**Education level; *P*<.001**					
	No education	40 (1.7)		3579 (24.2)	
	Primary school or lower	561 (24.3)		6907 (46.6)	
	Middle school	803 (34.7)		3016 (20.4)	
	High school or higher	908 (39.3)		1303 (8.8)	
**Nationality; *P*=.22**					
	Han	2168 (93.8)		13,780 (93.1)	
	Ethnic minorities	144 (6.2)		1025 (6.9)	
**Work; *P*<.001**					
	Yes	1600 (69.2)		9598 (64.8)	
	No/retired	712 (30.8)		5207 (35.2)	
**Age (years); *P*<.001**					
	45-54	1218 (52.7)		3515 (23.8)	
	55-64	787 (34.0)		5094 (34.4)	
	65-74	252 (10.9)		4400 (29.7)	
	≥75	55 (2.4)		1796 (12.1)	
**Area; *P*<.001**					
	Urban	967 (41.8)		2434 (16.4)	
	Rural	1345 (58.2)		12,371 (83.6)	
**Marital status; *P*<.001**					
	Married	2146 (92.8)		12,624 (85.3)	
	Unmarried	166 (7.2)		2181 (14.7)	
**Health insurance; *P*=.005**					
	Yes	2267 (98.1)		14,357 (97.0)	
	No	45 (1.9)		448 (3.0)	
**Smoking status; *P*=.009**					
	Yes	684 (29.6)		3993 (27.0)	
	No	1628 (70.4)		10,812 (73.0)	
**Drinking status; *P*<.001**					
	Yes	1191 (51.5)		4741 (32.0)	
	No	1121 (48.5)		10,064 (68.0)	
**Restless sleep times; *P*<.001**					
	Rare	1159 (50.1)		6674 (45.1)	
	Some or a little	469 (20.3)		2334 (15.8)	
	Occasionally	319 (13.8)		2316 (15.6)	
	Mostly or all the time	365 (15.8)		3481 (23.5)	
**ADL^a^; *P*<.001**					
	Unimpaired	2180 (94.3)		11,923 (80.5)	
	Impaired	132 (5.7)		2882 (19.5)	

^a^ADL: activity of daily living.

**Table 2:** Logistic regression analysis of internet use and internet use frequency among middle-aged and older adults.

Characteristics and variables			Internet use^a^			Internet use frequency^b^		
		OR^c^ (95% CI)		*P* value		OR (95% CI)	*P* value	
Gender (men vs women)			1.15 (1.00-1.31)		.04	1.18 (1.04-1.35)		.005
**Education (vs no education)**								
	Primary school or lower	4.76 (3.47-6.70)		<.001		4.96 (3.62-6.99)	<.001	
	Middle school	11.95 (8.71-16.86)		<.001		12.76 (9.31-17.99)	<.001	
	High school or higher	30.06 (21.80-42.62)		<.001		32.01 (23.24-45.34)	<.001	
Nationality (Han vs ethnic minorities)			—		—	—		—
Work (working vs not working/retired)			1.16 (1.03-1.32)		.02	—		—
**Age (vs 45-54 years)**								
	55-64	0.37 (0.33-0.42)		<.001		0.38 (0.34-0.42)	<.001	
	65-74	0.21 (0.18-0.25)		<.001		0.21 (0.18-0.25)	<.001	
	≥75	0.10 (0.07-0.14)		<.001		0.11 (0.08-0.14)	<.001	
Area (urban vs rural)			0.44 (0.40-0.50)		<.001	0.42 (0.38-0.47)		<.001
Marital status (married vs unmarried)			—		—	—		—
Health insurance (insured vs uninsured)			—		—	—		—
Smoking status (smoking vs not smoking)			1.15 (1.01-1.31)		.03	1.14 (1.01-1.30)		.02
Drinking status (drinking vs not drinking)			0.57 (0.51-0.63)		<.001	0.57 (0.51-0.64)		<.001
**Restless sleep times (vs rare)**								
	Some or a little	1.06 (0.93-1.21)		.37		1.03 (0.90-1.18)	.33	
	Occasionally	0.90 (0.77-1.04)		.15		0.85 (0.73-0.99)	.02	
	Mostly or all the time	0.86 (0.75-0.99)		.04		0.82 (0.72-0.94)	.003	
ADL^d^ (unimpaired vs impaired)			0.55 (0.45-0.67)		<.001	—		—

^a^Based on univariate analysis and stepwise regression screening, all sociodemographic factors except nationality, marital status, and health insurance were included in the binary logistic regression analysis for internet use.

^b^After stepwise regression screening and eliminating variables that caused collinearity, all sociodemographic factors except nationality, work, marital status, health insurance, and ADLs were included in the ordinal logistic regression analysis for internet use frequency.

^c^OR: odds ratio.

^d^ADL: activity of daily living.

## Discussion

### Principal Findings

Our work focused on assessing whether there is a correlation between daily internet use and the incidence of chronic diseases, in contrast to past studies that have focused on eHealth and mHealth. This longitudinal study reveals a relationship between daily internet use and a gradual decline in the incidence of numerous chronic diseases among middle-aged and older Chinese people and shows that the risk for some chronic diseases decreases as internet use frequency increases.

Daily internet use is not as purposeful as eHealth and mHealth in terms of obtaining health knowledge and health services, but it is, nevertheless, how most older adults access the internet, using it a social and entertainment medium [38]. A few factors support our hypothesis that daily internet use may lower the chances of developing certain chronic diseases.

First, middle-aged and older adults’ physical and mental health may be directly affected by the internet because through its effect on the volume of the globus pallidus, internet use is associated with positive effects on cognitive function [64]. Previous studies have reported that digital gameplay for older adults may slow cognitive decline [65,66]. In addition, there is ample proof that regular internet use, such as social interaction and communication, may satisfy people’s needs for information and social interaction, significantly lessen loneliness for individuals, improve psychological well-being, and benefit the mental health of middle-aged and older adults [42,67,68]. Moreover, online entertainment, such as watching videos and playing mobile games using the internet, may bring users more happiness and further improve life satisfaction [69,70].

Second, middle-aged and older adults are more concerned with their health and may be more inclined to use the internet regularly to obtain the health information they need to positively modify their behavior [5,71]. Well-established evidence shows that an unhealthy lifestyle accounts for more than two-thirds of chronic diseases [72]; therefore, changing one’s unhealthy lifestyle using the internet may prevent these diseases. For example, in groups at higher risk for cardiovascular disease, internet use may reduce the risk by encouraging them to adopt a balanced diet and physical exercise [73]. New prospects for the dissemination of online health information are presented through short videos and papers on official accounts and search engines with low-threshold access and high interaction. For instance, short health videos distributed through social media may influence middle-aged and older adults’ everyday activities and social interactions, providing them with health and medical information more efficiently and easily [74]. Notably, to prevent the spread of false health information, excessive entertainment, and other issues, the popularization of health knowledge in this way is still in progress and must be improved from the perspectives of content, form, and oversight mechanisms [75].

Finally, sharing through forwarding or recounting is a hallmark of daily internet use at the interpersonal level [74]. Chatting over the internet may boost the interactions and communication between middle-aged and older adults, enhancing their interpersonal relationships, which may subsequently have a direct or indirect impact on their health-related behaviors and status [76]. By exposing middle-aged and older adults to environments with web-based health knowledge dissemination, sharing health information may expand the reach of influence and make them more likely to participate in these initiatives and subsequently receive preventive intervention, which is challenging to do in other ways.

Nevertheless, in addition to the aforementioned reasons, there are several factors that may influence the occurrence of chronic diseases. For example, the dopamine transporter (*DAT1*) gene has been implicated in addiction, including excessive internet use [77]. This may indicate that internet use may affect the physical condition of the elderly through dopamine levels. Internet use may also be a marker of higher income levels for individuals and groups, which is related to better health care access and healthy lifestyles [78]. Moreover, internet use also indicates a level of improvement of people’s living environments. This may affect their health through direct passive impact, such as effects of indoor environmental quality or by influencing behaviors that can affect health [79]. However, the specific pathways of these influencing factors remain to be further studied.

Previous research has revealed that frequency, rather than the time spent, may be a better measure of the motive for using the internet [80]. Our study showed that with the increasing frequency of internet use, the risk of some chronic diseases decreases. This may be because more frequent internet use can increase life satisfaction and enhance the quality of life [43,81]. Previous studies have also found that more frequent internet use is related to easier adoption of preventive behaviors [82]. In addition, studies have shown that more frequent internet use is positively related to the overall preferences for obtaining health information and decision-making autonomy associated with higher health literacy levels [21,83,84]. However, excessive internet use could also have a negative impact on health [85,86]. For instance, Liao et al [58] found that overuse of the internet may be associated with more severe depression. Therefore, although we believe that frequent internet use may further lower the risk for certain chronic diseases, it is also important to maintain good online behaviors, notably avoiding using the internet for extended periods of time.

Internet use among middle-aged and older adults is linked to socioeconomic and demographic characteristics. Our study supports previous studies showing that middle-aged and older adults who are women, young, educated, unemployed, and residing in urban areas are more likely to use the internet because they accept it more [5,45,87]. It should be noted that in this study, the results of univariate analyses of gender, work, and area were not consistent with the results of multivariate analysis, which may be due to the effects of other confounding factors. Physical issues that make learning digital skills difficult may also prevent older adults from using the internet [88]. Similarly, restless sleep times and impaired ADLs have been attributed to internet use. Consistent with Duplaga’s and Peng’s results [89,90], internet use is associated with more frequent drinking (implying more social opportunities) and the healthier habit of less frequent smoking. Studying the factors that influence middle-aged and older adults using the internet may help us better understand the challenges they face and improve the internet’s capacity to slow the progression of chronic diseases.

In this study, 12.3% middle-aged and older adults in China used the internet, comparable to that reported by Lyu and Sun [91] in China but lower than that reported in high-income countries [46], which may result from varied levels of economic progress. Middle-aged and older people have steadily increased and maintained a high level of internet use in recent years, and over half of the respondents use the internet every day [5]. It is interesting to note that older adults used the internet more regularly. This may due to the rapid popularization of the internet and the fact that retired people have more free time than middle-aged people. This pattern suggests that access to reliable and scientific health information over the internet may significantly improve the health of middle-aged and older adults. Given the differences in daily internet use between urban and rural areas, governments should strengthen the network infrastructure and increase the equality in internet access among older people. Studies have found that middle-aged and older adults living in rural areas have fewer medical and health resources than those living in metropolitan areas, possibly exacerbating the health gap acquired through the internet. Elderly individuals in rural areas may thus benefit more from increased internet aid [51].

If future research further validates the causal relationship between daily internet use and chronic diseases, alternative strategies may be considered to promote daily internet use among middle-aged and older adults, considering the distinctive qualities of each group. Family digital feedback may be implemented to encourage the younger members of the family to teach older people about digital technology and stimulate their interest in the internet. Considering older people have high dependence on and trust in traditional media, such as television and newspapers, these channels can also be used for publicity [92,93]. Additionally, internet service providers should make efforts to improve themselves to fulfill the requirements of middle-aged and older adults who use the internet on a daily basis. For instance, they should use charts rather than large fonts and include operation prompts, voice aid, and other features to make the presented material as simple and user friendly as possible to improve the use of internet devices among older people [93]. Meanwhile, to address the challenge of combating false information during daily internet use, a range of approaches may be used, including technical measures, such as artificial intelligence algorithms for authenticity verification, timely official government surveillance, and public education on how to critically evaluate the credibility of internet-based information [75,93-95]. However, privacy concerns must also be considered when implementing such measures [93].

### Strengths and Limitations

The study offers several advantages. To thoroughly explore the association between daily internet use and the incidence of chronic diseases and provide empirical evidence in favor of preventing rather than treating chronic diseases, middle-aged and older adults without chronic diseases were our target group. Second, in this paper, we chose to focus on daily internet use among older populations, which has more practical implications for the prevention of chronic diseases. Four waves of CHARLS cohort data were included in this study, which represents the relationship between daily internet use and the risk for chronic diseases in middle-aged and older adults more accurately, thoroughly, and precisely over time.

There are several limitations to be aware of in this study. First, the sample was restricted to Chinese participants due to data availability, which may limit the generalizability of the findings to other countries or populations. Additionally, the use of self-reported data for quantification, such as chronic disease status, may be subject to reporting bias, which could affect the accuracy of the results. Furthermore, the study did not control for sociodemographic factors, such as income and living environment, or clinical metrics, such as genetics, which may be related to the occurrence of chronic diseases. Lastly, detailed information about the participants’ daily internet use, such as internet access and internet characteristics, was not collected. It is imperative to conduct future prospective research, such as randomized controlled trials and large cohort studies, to authenticate the findings. These studies should consider controlling for these additional factors when exploring the effects of different patterns of daily internet use on middle-aged and older adults.

### Conclusion

USING the CHARLS survey, this study found that middle-aged and elderly Chinese people who use the internet daily have a reduced risk of developing chronic diseases and provided specific recommendations on how to improve daily internet use. With middle-aged and older adults using the internet more than ever before in China, it has gradually become a near necessity of their daily life. The findings illustrate that promoting safe and appropriate daily internet use may be a potential strategy for improving health outcomes for adults, but further robust and prospective studies are reqiured to validate the findings prior to implementing corresponding measures.

## Appendix

Multimedia Appendix 1 Supplementary tables and figures for the study.

## Abbreviations

ADL: activity of daily living
CAPI: computer-assisted personal interview
CES-D10: Center for Epidemiologic Studies Depression Scale
CHARLS: China Health and Retirement Longitudinal Study
HR: hazard ratio
mHealth: mobile health
OR: odds ratio
VI: vision impairment

## Data Availability

The data sets used and analyzed during the study are available [96].
