# Supplementary material for: Association Between Daily Internet Use and Incidence of Chronic Diseases Among Older Adults: Prospective Cohort Study
Source: J Med Internet Res. 2023 Jul 17;25:e46298. doi: 10.2196/46298 (PMC10390981; doi:10.2196/46298)

**Multimedia Appendix**

**Supplemental Table S1.** Survey questions and classification of variables.

**Supplemental Table S2.** Characteristics of participants by daily internet use frequency.

**Supplemental Table S3.** Unadjusted models and adjusted models estimating the association between daily internet use and the incidence of chronic diseases.

**Supplemental Table S4.** Unadjusted models and adjusted models estimating the association between daily internet use frequency and the incidence of chronic diseases.

**Supplemental Figure S1.** Gender difference of daily internet use among middle-aged and older adults in different age groups: CHARLS 2011-2018. **P*<.05.

**Supplemental Figure S2.** Gender difference of daily internet use frequency among middle-aged and older adults in different age groups: CHARLS 2011-2018. Freq 1 = using internet not frequently, Freq 2 = using internet almost every week, Freq 3 = using internet almost daily. **P*<.05.

**Supplemental Table S1.** Survey questions and classification of variables.

| **Variable** | **Questions** | **Score** | **Classification** |
| --- | --- | --- | --- |
| ADL^a^ | "Do you have any trouble dressing?"  "Do you have any trouble bathing or showering?"  "Do you have any trouble eating?"  "Do you have any trouble getting into or out of bed?"  "Do you have any trouble using the toilet, including getting up?"  "Do you have any trouble regulating urine and defecation?" | 1 = I don’t have any difficulty  2 = I have difficulty but can still do it  3 = I have difficulty and need help  4 = I cannot do it | 6 indicates healthy;  7 to 9 indicates mild disability;  10 to 14 indicates moderate disability;  15 to 19 indicates partial disability;  20 to 24 indicates severe disability;  “healthy” was considered as unimpaired.  “mild disability, moderate disability, partial disability and severe disability” were considered as impaired. |
| Depression | "I was bothered by things that don’t usually bother me"  "I had trouble keeping on things"  "I felt depressed"  "I felt everything I did was an effort"  "I felt hopeful about the future^b^"  "I felt fearful"  "My sleep was restless"  "I was happy^b^"  "I felt lonely"  "I could not get going" | 0 = less than 1 day,  1 = 1-2 days,  2 = 3-4 days,  3 = 5-7 days. | A cut-off score of 10 was used to identify risk of depression. |
| Vision impairment | "Do you have vision problem (blind or partially blind)?"  "Do you usually wear glasses or corrective lenses? "  "How good is your eyesight for seeing things at a distance? "  "How good is your eyesight for seeing things up close? "  "Have you ever been treated for cataract? "  "Have you ever been treated for glaucoma? " | / | If participants reported “Have vision problems, usually wear glasses or corrective lenses, poor eyesight for seeing things at a distance or seeing things up close, have cataracts or have glaucoma”, they were classified as having vision impairment. |

^a^ADL: activities of daily living.

^b^Two positively worded items (hopeful and happy) were reverse coded before analysis.

**Supplemental Table S2.** Characteristics of participants by daily internet use frequency.

| **Variables** | **Internet use frequency**^a^ | | | | ***P* value** |
| --- | --- | --- | --- | --- | --- |
|  | 0  (n=14,805) | 1  (n=219) | 2  (n=175) | 3  (n=1,918) |  |
| **Gender, n (%)** |  |  |  |  | <.001 |
| Men | 6902 (46.6) | 127 (58.0) | 104 (59.4) | 1056 (55.1) |  |
| Women | 7903 (53.4) | 92 (42.0) | 71 (40.6) | 862 (44.9) |  |
| **Education level, n (%)** |  |  |  |  | <.001 |
| No education | 3579 (24.2) | 9 (4.1) | 5 (2.9) | 26 (1.4) |  |
| Primary school or lower | 6907 (46.6) | 69 (31.5) | 56 (32.0) | 436 (22.7) |  |
| Middle school | 3016 (20.4) | 86 (39.3) | 48 (27.4) | 669 (34.9) |  |
| High school or higher | 1303 (8.8) | 55 (25.1) | 66 (37.7) | 787 (41.0) |  |
| **Nationality, n (%)** |  |  |  |  | .03 |
| Han | 13,780 (93.1) | 197 (90.0) | 160 (91.4) | 1811 (94.4) |  |
| Ethnic minorities | 1025 (6.9) | 22 (10.0) | 15 (8.6) | 107 (5.6) |  |
| **Work, n (%)** |  |  |  |  | <.001 |
| Yes | 9598 (64.8) | 168 (76.7) | 124 (70.9) | 1308 (68.2) |  |
| No/retired | 5207 (35.2) | 51 (23.3) | 51 (29.1) | 610 (31.8) |  |
| **Age(years), n (%)** |  |  |  |  | <.001 |
| 45-54 | 3515 (23.8) | 132 (60.3) | 90 (51.4) | 996 (51.9) |  |
| 55-64 | 5094 (34.4) | 66 (30.1) | 66 (37.7) | 655 (34.2) |  |
| 65-74 | 4400 (29.7) | 20 (9.1) | 16 (9.2) | 216 (11.3) |  |
| ≥75 | 1796 (12.1) | 1 (0.5) | 3 (1.7) | 51 (2.6) |  |
| **Area, n (%)** |  |  |  |  | <.001 |
| Urban | 2434 (16.4) | 56 (25.6) | 57 (32.6) | 854 (44.5) |  |
| Rural | 12,371 (83.6) | 163 (74.4) | 118 (67.4) | 1064 (55.5) |  |
| **Marital status, n (%)** |  |  |  |  | <.001 |
| Married | 12,624 (85.3) | 209 (95.4) | 161 (92.0) | 1776 (92.6) |  |
| Unmarried | 2181 (14.7) | 10 (4.6) | 14 (8.0) | 142 (7.4) |  |
| **Health insurance, n (%)** |  |  |  |  | .04 |
| Yes | 14,357 (97.0) | 215 (98.2) | 172 (98.3) | 1880 (98.0) |  |
| No | 448 (3.0) | 4 (1.8) | 3 (1.7) | 38 (2.0) |  |
| **Smoking status, n (%)** |  |  |  |  | .04 |
| Yes | 3993 (27.0) | 73 (33.3) | 50 (28.6) | 561 (29.2) |  |
| No | 10,812 (73.0) | 146 (66.7) | 125 (71.4) | 1357 (70.8) |  |
| **Drinking status, n (%)** |  |  |  |  | <.001 |
| Yes | 4741 (32.0) | 115 (52.5) | 96 (54.9) | 980 (51.1) |  |
| No | 10,064 (68.0) | 104 (47.5) | 79 (45.1) | 938 (48.9) |  |
| **Restless sleep times, n (%)** |  |  |  |  | <.001 |
| Rare | 6674 (45.1) | 109 (49.8) | 76 (43.4) | 974 (50.8) |  |
| Some or a little | 2334 (15.8) | 45 (20.5) | 38 (21.7) | 386 (20.1) |  |
| Occasionally | 2316 (15.6) | 30 (13.7) | 36 (20.6) | 253 (13.2) |  |
| Most or all the time | 3481 (23.5) | 35 (16.0) | 25 (14.3) | 305 (15.9) |  |
| **ADL**^b^**, n (%)** |  |  |  |  | <.001 |
| Unimpaired | 11,923 (80.5) | 203 (92.7) | 162 (92.6) | 1815 (94.6) |  |
| Impaired | 2882(19.5) | 16 (7.3) | 13 (7.4) | 103 (5.4) |  |

^a^0 = not using internet, 1 = using Internet not frequently, 2 = using internet almost every week, 3 = using internet almost daily.

^b^ADL: activities of daily living.

**Supplemental Table S3.** Unadjusted models and adjusted models estimating the association between daily internet use and the incidence of chronic diseases.

| **Disease** | **Model 1^a^** | | **Model 2^b^** | | **Model 3^c^** | |
| --- | --- | --- | --- | --- | --- | --- |
|  | **HR (95% CI)** | ***P* value** | **HR (95% CI)** | ***P* value** | **HR (95% CI)** | ***P* value** |
| Hypertension | 0.63 (0.53 to 0.75) | **<.001** | 0.78 (0.64 to 0.94) | **.01** | 0.78 (0.65 to 0.95) | **.01** |
| Dyslipidemia | 1.08 (0.92 to 1.26) | .35 | 0.97 (0.82 to 1.14) | .70 | 0.99 (0.84 to 1.18) | .95 |
| Diabetes | 0.76 (0.61 to 0.96) | **.02** | 0.81 (0.63 to 1.04) | .10 | 0.84 (0.66 to 1.07) | .17 |
| Cancer | 0.88 (0.59 to 1.31) | .53 | 0.95 (0.62 to 1.46) | .83 | 0.97 (0.63 to 1.50) | .90 |
| Chronic lung disease | 0.55 (0.43 to 0.71) | **<.001** | 0.70 (0.54 to 0.92) | **.009** | 0.74 (0.57 to 0.97) | **.03** |
| Liver disease | 1.12 (0.88 to 1.42) | .37 | 1.04 (0.80 to 1.36) | .75 | 1.08 (0.83 to 1.41) | .56 |
| Heart disease | 0.76 (0.62 to 0.92) | .006 | 0.80 (0.65 to 0.98) | **.03** | 0.82 (0.66 to 1.01) | .06 |
| Stroke | 0.54 (0.40 to 0.73) | **<.001** | 0.64 (0.47 to 0.88) | **.006** | 0.69 (0.50 to 0.94) | **.02** |
| Kidney disease | 0.79 (0.62 to 1.01) | .06 | 0.83 (0.64 to 1.08) | .16 | 0.88 (0.68 to 1.15) | .36 |
| Digestive disease | 0.59 (0.48 to 0.74) | **<.001** | 0.70 (0.56 to 0.87) | **.002** | 0.73 (0.58 to 0.91) | **.005** |
| Emotional and psychiatric disease | 0.41 (0.24 to 0.69) | **<.001** | 0.55 (0.32 to 0.97) | **.04** | 0.61 (0.35 to 1.07) | .09 |
| Memory-related disorders | 0.40 (0.26 to 0.62) | **<.001** | 0.54 (0.35 to 0.84) | **.007** | 0.58 (0.37 to 0.91) | **.02** |
| Arthritis or rheumatism | 0.43 (0.35 to 0.54) | **<.001** | 0.59 (0.47 to 0.74) | **<.001** | 0.60 (0.48 to 0.76) | **<.001** |
| Asthma | 0.32 (0.20 to 0.50) | **<.001** | 0.49 (0.31 to 0.79) | **.003** | 0.52 (0.33 to 0.84) | **.007** |
| Depression | 0.73 (0.65 to 0.80) | **<.001** | 0.78 (0.70 to 0.87) | **<.001** | 0.80 (0.71 to 0.89) | **<.001** |
| Vision impairment | 0.74 (0.66 to 0.82) | **<.001** | 0.82 (0.73 to 0.92) | **<.001** | 0.83 (0.74 to 0.93) | **.004** |

^a^Model 1 was a univariate model of internet use.

^b^Model 2 was adjusted for socioeconomic factors, such as gender, education, nationality, work status, recruitment age, resident area, marital status, and health insurance.

^c^Model 3 was the final parsimonious model, further adjusted for health behaviors, including smoking status, drinking status, restless sleep times, and activities of daily living (ADL).

**Supplemental Table S4.** Unadjusted models and adjusted models estimating the association between daily internet use frequency and the incidence of chronic diseases.

| **Disease** | **Model 1^a^** | | **Model 2^b^** | | **Model 3^c^** | |
| --- | --- | --- | --- | --- | --- | --- |
|  | **HR (95% CI)** | ***P* value** | **HR (95% CI)** | ***P* value** | **HR (95% CI)** | ***P* value** |
| Hypertension | 0.84 (0.78 to 0.90) | **<.001** | 0.91 (0.85 to 0.98) | **.01** | 0.91 (0.85 to 0.98) | **.01** |
| Dyslipidemia | 1.05 (0.99 to 1.11) | .11 | 1.01 (0.95 to 1.08) | .74 | 1.02 (0.96 to 1.09) | .50 |
| Diabetes | 0.89 (0.82 to 0.98) | **.01** | 0.91 (0.83 to 1.00) | .06 | 0.93 (0.84 to 1.02) | .11 |
| Cancer | 0.94 (0.81 to 1.10) | .45 | 0.97 (0.82 to 1.14) | .69 | 0.97 (0.82 to 1.15) | .75 |
| Chronic lung disease | 0.81 (0.74 to 0.89) | **<.001** | 0.89 (0.81 to 0.99) | **.03** | 0.91 (0.83 to 1.01) | .70 |
| Liver disease | 1.04 (0.95 to 1.14) | .43 | 1.01 (0.91 to 1.12) | .84 | 1.02 (0.93 to 1.13) | .63 |
| Heart disease | 0.92 (0.85 to 0.99) | **.02** | 0.93 (0.86 to 1.01) | .08 | 0.94 (0.87 to 1.02) | .14 |
| Stroke | 0.82 (0.73 to 0.91) | **<.001** | 0.87 (0.78 to 0.98) | **.03** | 0.89 (0.79 to 1.01) | .07 |
| Kidney disease | 0.91 (0.82 to 1.00) | **.045** | 0.92 (0.83 to 1.02) | .11 | 0.94 (0.85 to 1.05) | .27 |
| Digestive disease | 0.81 (0.75 to 0.88) | **<.001** | 0.86 (0.79 to 0.94) | **<.001** | 0.87 (0.80 to 0.95) | **.003** |
| Emotional and psychiatric disease | 0.68 (0.54 to 0.85) | **<.001** | 0.76 (0.61 to 0.96) | **.02** | 0.79 (0.63 to 1.00) | .05 |
| Memory-related disorders | 0.73 (0.62 to 0.85) | **<.001** | 0.81 (0.69 to 0.96) | **.01** | 0.83 (0.71 to 0.99) | **.03** |
| Arthritis or rheumatism | 0.72 (0.66 to 0.78) | **<.001** | 0.81 (0.74 to 0.89) | **<.001** | 0.82 (0.75 to 0.89) | **<.001** |
| Asthma | 0.65 (0.54 to 0.78) | **<.001** | 0.77 (0.64 to 0.93) | **.006** | 0.79 (0.66 to 0.95) | **.01** |
| Depression | 0.89 (0.85 to 0.92) | **<.001** | 0.91 (0.87 to 0.95) | **<.001** | 0.92 (0.88 to 0.96) | **<.001** |
| Vision impairment | 0.89 (0.86 to 0.93) | **<.001** | 0.93 (0.89 to 0.97) | **.002** | 0.94 (0.89 to 0.98) | **.004** |

^a^Model 1 was a univariate model of internet use.

^b^Model 2 was adjusted for socioeconomic factors, such as gender, education, nationality, work status, recruitment age, resident area, marital status, and health insurance.

^c^Model 3 was the final parsimonious model, further adjusted for health behaviors, including smoking status, drinking status, restless sleep times, and activities of daily living (ADL).

**Supplemental Figure S1.** Gender difference of daily internet use among middle-aged and older adults in different age groups: CHARLS 2011-2018. **P*<.05.


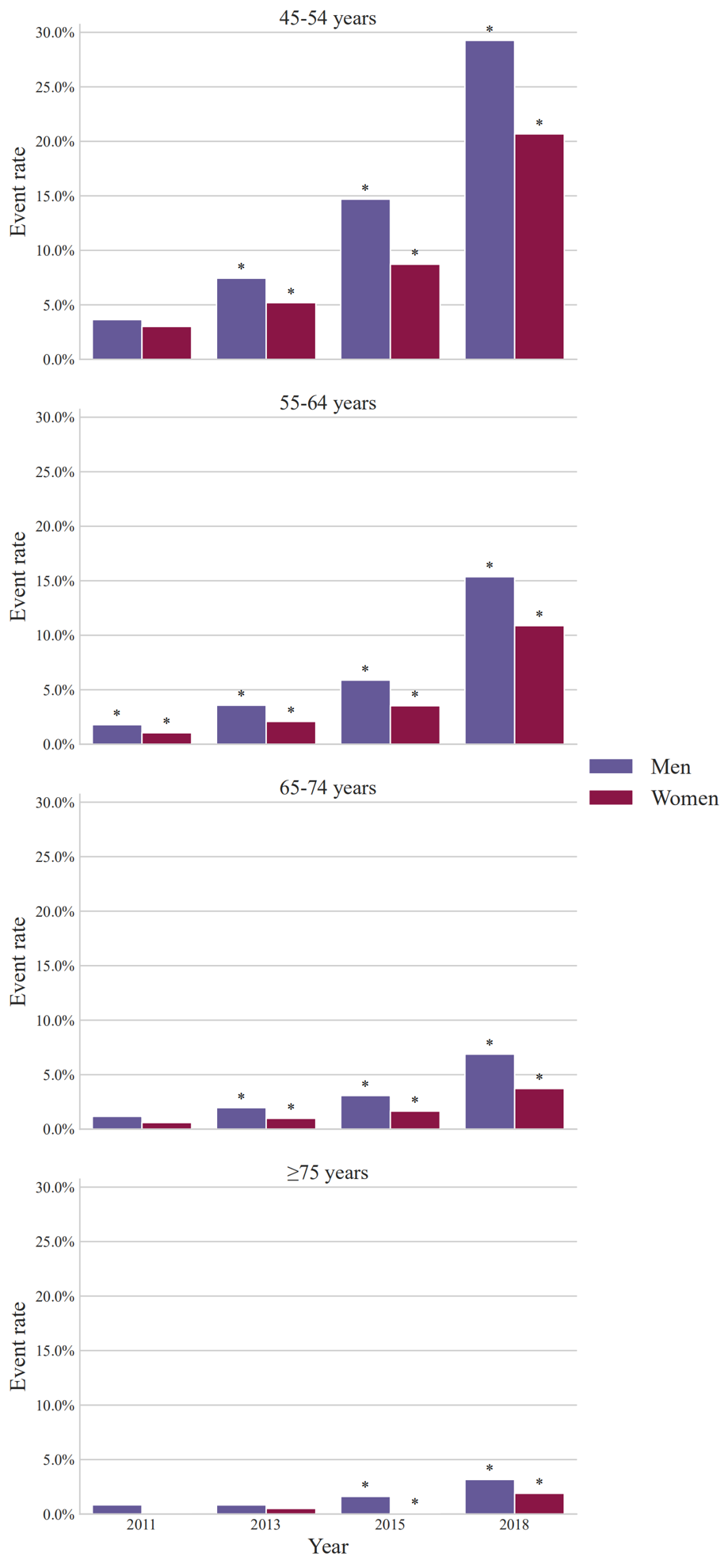


**Supplemental Figure S2.** Gender difference of daily internet use frequency among middle-aged and older adults in different age groups: CHARLS 2011-2018. Freq 1 = using internet not frequently, Freq 2 = using internet almost every week, Freq 3 = using internet almost daily. **P*<.05.


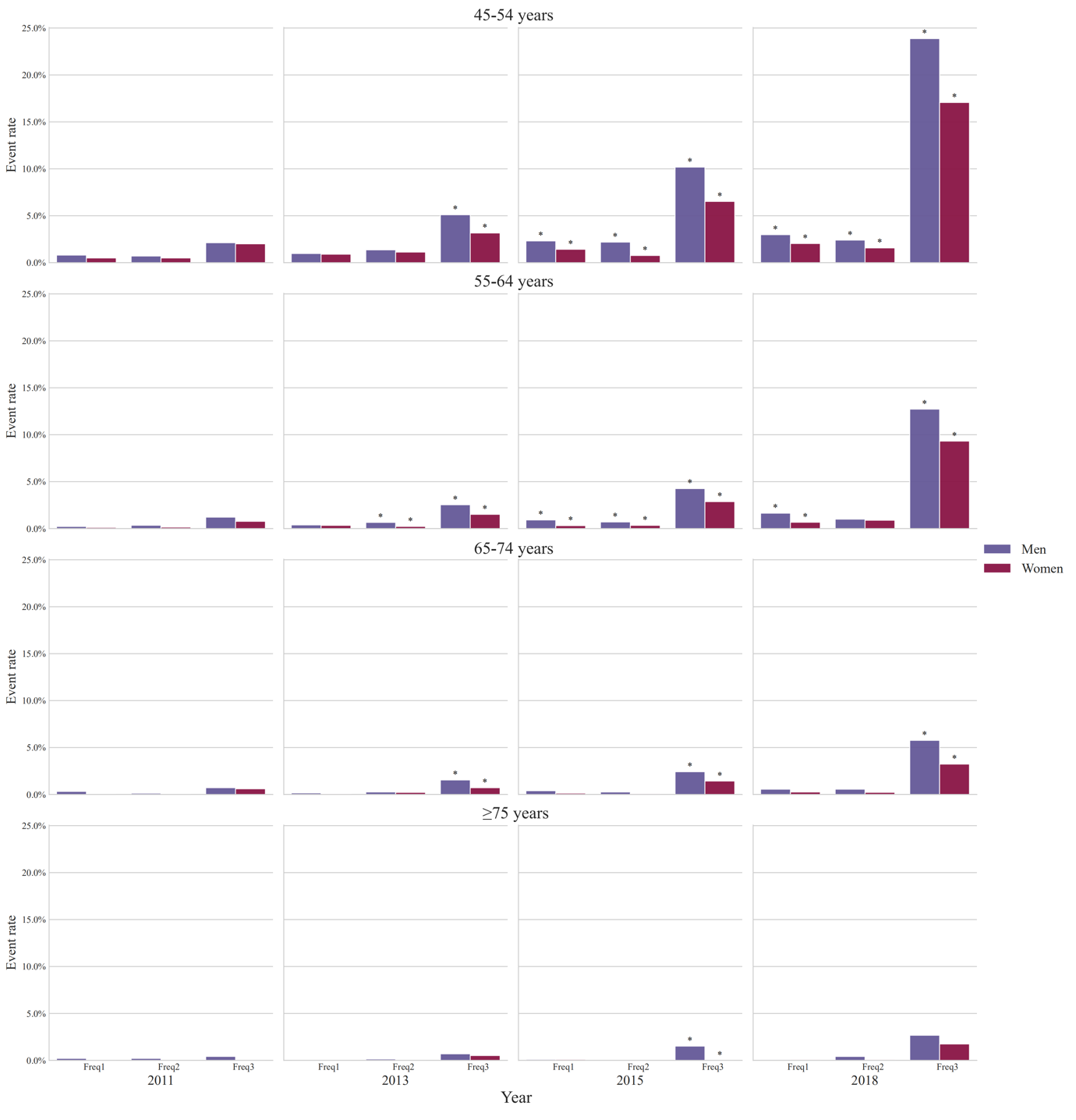

Supplement: Multimedia Appendix 1 [file jmir_v25i1e46298_app1.docx]
